# Validation of the 2023 International Diagnostic Criteria for MOGAD in a Selected Cohort of Adults and Children

**DOI:** 10.1212/WNL.0000000000209321

**Published:** 2024-06-13

**Authors:** James A. Varley, Dimitrios Champsas, Timothy Prossor, Giuseppe Pontillo, Omar Abdel-Mannan, Zhaleh Khaleeli, Axel Petzold, Ahmed T. Toosy, Sachid A. Trip, Heather Wilson, Dermot H. Mallon, Cheryl Hemingway, Kshitij Mankad, Michael Kin Loon Chou, Andrew J. Church, Melanie S. Hart, Michael P. Lunn, Wallace Brownlee, Yael Hacohen, Olga Ciccarelli

**Affiliations:** From the Queen Square MS Centre (J.A.V., G.P., O.A.-M., Z.K., A.P., A.T.T., S.A.T., H.W., D.H.M., C.H., M.S.H., W.B., Y.H., O.C.), Department of Neuroinflammation, UCL Queen Square Institute of Neurology, University College London; Department of Brain Sciences (J.A.V.), Charing Cross Hospital, Imperial College London; Department of Neurology (D.C., O.A.-M., C.H., Y.H.), Great Ormond Street Hospital for Children; The National Hospital for Neurology and Neurosurgery (T.P., Z.K., A.P., A.T.T., S.A.T., H.W., D.H.M., M.S.H., M.P.L., W.B., O.C.), UCLH NHS Trust, London, United Kingdom; Neuro-ophthalmology Expert Centre (A.P.), Amsterdam UMC, the Netherlands; Moorfields Eye Hospital NHS Foundation Trust (A.P.); Department of Radiology (K.M.), Great Ormond Street Hospital for Children; Neuroimmunology and CSF Laboratory (M.K.L.C., A.J.C., M.S.H., M.P.L.), National Hospital for Neurology and Neurosurgery; National Institute for Health and Care Research (NIHR) (M.S.H., M.P.L., W.B., O.C.), University College London Hospitals Biomedical Research Centre; and Department of Neuromuscular Diseases (M.P.L.), UCL Queen Square Institute of Neurology, University College London, United Kingdom.

## Abstract

**Background and Objectives:**

To test the performance of the 2023 myelin oligodendrocyte glycoprotein antibody–associated disease (MOGAD) criteria in adults and children with inflammatory demyelinating conditions who were tested for MOG antibodies (Abs).

**Methods:**

This was a retrospective study of patients tested for MOG-Abs from 2018 to 2022 in 2 specialist hospitals. The inclusion criteria comprised ≥1 attendance in an adult or pediatric demyelinating disease clinic and complete clinical and MRI records. The final clinical diagnosis of MOGAD, made by the treating neurologist, was taken as the benchmark against which the new criteria were tested. The international MOGAD diagnostic criteria were applied retrospectively; they stipulate at least 1 clinical or MRI supporting feature for MOGAD diagnosis in positive fixed MOG cell-based assay without a titer. The performance MOG-Ab testing alone for MOGAD diagnosis was also assessed and compared with that of MOGAD criteria using the McNemar test.

**Results:**

Of the 1,879 patients tested for MOG-Abs, 539 (135 pediatric and 404 adults) met the inclusion criteria. A clinical diagnosis of MOGAD was made in 86/539 (16%) patients (37 adults, 49 children), with a median follow-up of 3.6 years. The MOGAD diagnostic criteria had sensitivity of 96.5% (adults 91.9%, children 100%), specificity of 98.9% (adults 98.8%, children 98.9%), positive predictive value of 94.3% (adults 89.4%, children 98%), negative predictive value of 99.3% (adults 99.2%, children 100%), and accuracy of 98.5% (adults 98.3%, children 99.2%). When compared with MOG-Ab testing alone, a difference was seen only in adults: a significantly higher specificity (98.9% vs 95.6%, *p* = 0.0005) and nonstatistically significant lower sensitivity (91.9% vs 100%, *p* = 0.08).

**Discussion:**

The international MOGAD diagnostic criteria exhibit high performance in selected patients with inflammatory demyelinating diseases (who had a high pretest probability of having MOGAD) compared with best clinical judgment; their performance was better in children than in adults. In adults, the MOGAD criteria led to an improvement in specificity and positive predictive value when compared with MOG-Ab testing alone, suggesting that the requirement of at least 1 clinical or MRI supporting feature is important. Future work should address the generalizability of the diagnostic criteria to cohorts of greater clinical diversity seen within neurologic settings.

## Introduction

Myelin oligodendrocyte glycoprotein antibody (MOG-Ab)–associated disease (MOGAD) is a recognized demyelinating disease distinct from multiple sclerosis (MS) and aquaporin-4 antibody (AQP4-Ab) neuromyelitis optica spectrum disorder (NMOSD).^1-5^ International consensus criteria for the diagnosis of MOGAD have recently been published.^6^ These recommend that for a confirmed diagnosis of MOGAD, patients are required to have 1 core clinical phenotype (from optic neuritis, myelitis, acute disseminated encephalomyelitis [ADEM], cerebral monofocal or polyfocal deficits, brainstem/cerebellar deficits, cortical encephalitis) in combination with positive serum MOG Abs, either from a fixed cell-based assay (CBA) with titer ≥1:100 or a live CBA positive according to the standardized cutoff of that assay. If the antibody titer is unavailable or the result is low positive, then at least 1 supporting clinical or MRI feature is required for a MOGAD diagnosis^2^ (see list of supporting features in Table 1). Similar to the McDonald criteria for MS, the exclusion of alternative diagnoses is required, thereby posing challenges in clinical practice.^7^ The 2023 MOGAD criteria do not recommend MOG-Ab testing for screening of all patients with CNS inflammatory demyelination and advise that *typical* features of MOGAD should be seen before performing MOG-Ab testing.

**Table 1:** Supporting Clinical or MRI Features: At Least 1 Feature Needs to Be Present for a Diagnosis of MOGAD in Case of Low-Positive Titer or Positive Antibodies Without Reported Titer

Core clinical attacks	Supporting features
Optic neuritis	Bilateral simultaneous clinical involvement
	Longitudinal optic nerve involvement (>50% of the optic nerve length)
	Perineural optic sheath enhancement
	Optic disc edema
Myelitis	Longitudinal extensive myelitis
	Central cord lesion or H-sign
	Conus lesion
Brain, brainstem, or cerebral syndrome (including ADEM; cerebral monofocal or polyfocal deficits; brainstem or cerebellar deficits; cortical encephalitis)	Multiple ill-defined T2 hyperintense lesions in supratentorial and often infratentorial white matter
	Deep gray matter involvement
	Ill-defined T2 hyperintensity involving pons, middle cerebellar peduncle, or medulla
	Cortical lesion with or without lesional and overlying meningeal enhancement

Abbreviations: ADEM = acute disseminated encephalomyelitis; MOGAD = myelin oligodendrocyte glycoprotein antibody–associated disease.

Adapted from reference 6.

We aimed to test the performance of the 2023 international MOGAD diagnostic criteria in a retrospective cohort of patients with a suspected inflammatory demyelinating presentation, who were tested for MOG-Ab during their diagnostic workup, using the clinician diagnosis of MOGAD as the benchmark against which the new criteria were tested. We also investigated the performance of using MOG-Ab testing alone. We described the distribution of the supporting clinical and MRI features, which are required for a diagnosis of MOGAD according to 2023 criteria, according to phenotype, age, and onset vs final follow-up.

## Methods

This was a retrospective study of patients from the National Hospital for Neurology and Neurosurgery, UCLH NHS Trust, and Great Ormond Street Children's Hospital, GOSH NHS Trust, tested between August 20, 2018, and August 16, 2022, in the same laboratory (UCLH Neuroimmunology and CSF laboratory) for MOG-Ab. The inclusion criteria were as follows: (1) at least 1 encounter in a specialist adult or pediatric specialist neuroimmunology clinic for a suspected inflammatory demyelinating episode; (2) complete clinical data and all available brain ± spinal cord MRI records, collected at any time during the diagnostic workup (i.e., not always coincident with MOG-Ab testing); (3) at least 1 MOG-Ab test requested as part of diagnostic workup, during any of the patient's clinic appointments (i.e., not always at their first visit); and (4) MOG-Ab testing using fixed cell-based assay (Euroimmun fixed CBA), whose results were either negative or positive (i.e., without a titer during this study) by scientific staff experienced in CBA interpretation.

Clinical data, results of MOG-Ab testing, and brain and spinal cord MRI of patients who fulfilled the inclusion criteria were collected using hospital electronic health records. Patients were divided into groups on the basis of their presenting phenotype: optic neuritis; transverse myelitis; and brain, brainstem, or cerebral syndrome (including ADEM).

The final clinical diagnosis of MOGAD, made by the treating neurologist/pediatric neurologist at any of the patients' visits and confirmed at last follow-up, was taken as the diagnostic benchmark against which the new criteria were tested. These diagnoses were verified by Y.H. (all cases), J.V., A.T., and O.C. (adult cases). The performance of the 2023 MOGAD diagnostic criteria was tested by evaluating all MRI scans during acute attacks and all clinical information; the MOGAD diagnostic criteria require at least 1 clinical or MRI supporting feature (Table 1) in the presence of a positive fixed MOG-CBA without a titer. Sensitivity, specificity, accuracy, and positive predictive value (PPV) and negative predictive value (NPV) of the international MOGAD diagnostic criteria with 95% CI were calculated.^4,5^ These characteristics were obtained for both adults and children together and then separately for each cohort. Finally, the performance of MOG-Ab testing alone was assessed. The McNemar test was used to compare the MOGAD diagnostic criteria and MOG-Ab testing alone, by including adults and children, first together and then separately. In particular, the MOGAD and non-MOGAD groups were considered separately to explicitly compare their performances for sensitivity and specificity, respectively.^8^ To test the differences in the number of supporting clinical or MRI features between: (1) adults and children, (2) monophasic and relapsing disease, and (3) onset and final follow-up, parametric or nonparametric statistical tests (Mann-Whitney *U* and Kruskal-Wallis tests) were used for continuous distributions, as appropriate and χ^2^ or Fisher exact tests for nominal data. All results associated with a *p* value <0.05 were considered significant and reported.

### Standard Protocol Approvals, Registrations, and Patient Consents

This study was approved as an audit by GOSH Research and Development Department (reference: 16NC10) and as a service evaluation project by UCLH NHS Trust (15-202324-SE).

### Data Availability

For purposes of replicating procedures and results, a qualified investigator can request anonymized data after ethics clearance and approval by all authors.

## Results

Of the 1,879 patients tested for MOG-Ab, 539 patients met all inclusion criteria and were used to test the MOGAD criteria. The reasons why 1,340 were not included were as follows: (1) never seen in a specialist neuroimmunology clinic (N = 1,335) and (2) incomplete clinical and MRI data (N = 5).

**Figure 1:**
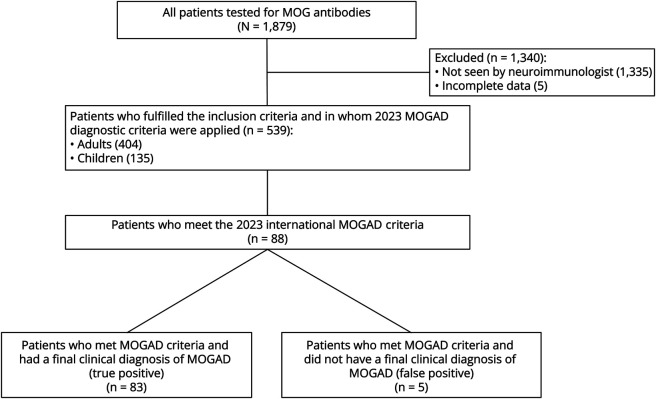
Flowchart Shows the Number of Patients Included In the Study, Patients With MOGAD Clinical Diagnosis, and Patients Who Meet the International MOGAD Criteria MOGAD = myelin oligodendrocyte glycoprotein antibody–associated disease.

Of these 539 patients, 103 (19%) tested positive for MOG-Ab using fixed CBA (53 adults and 50 children) (Figure 1) at a median of 3 months from symptom onset (range 0–30). MOGAD was the final diagnosis, based on best clinical judgment before the 2023 international diagnostic criteria, in 86/103 (83.6%) patients (37 adults, 49 children) (Figure 1); these 86 patients had been followed up in clinic for a median of 3.6 years (range 22–104 months), had a median age of 14 years (range 7 months–76 years), and had a slight female predominance (N = 48, 55.8%). The most common phenotype at presentation was optic neuritis (N = 40, 46.5%), followed by the brain, brainstem, and cerebral syndrome phenotype (N = 32, 37.2%) and then transverse myelitis (N = 14, 16.2%).

**Figure 2:**
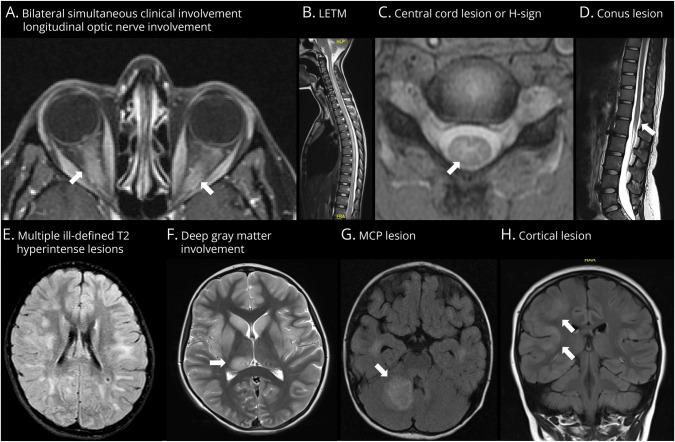
Radiologic Supporting Features (A) Axial gadolinium-enhanced fat sat T1-weighted scan of the brain showing longitudinal optic nerve involvement and bilateral simultaneous optic nerve involvement, which were the 2 most common supporting features in patients with predominant optic neuritis phenotypes. (B) Sagittal T2-weighted spinal cord scan showing a longitudinally extensive lesion in a patient with a predominant transverse myelitis phenotype. (C) Axial gradient echo T2-weighted scan of the cord showing the central lesion with a H-sign. (D) Sagittal T2-weighted spinal cord scan showing a conus lesion. (E) Axial FLAIR T2-weighted scan of the brain showing ill-defined lesions in a patient with brain, brainstem, or cerebral syndrome. (F) Axial T2-weighted scan of the brain showing a right thalamic lesion. (G) Axial FLAIR T2-weighted scan showing a lesion in the right middle cerebellar peduncle. (H) Coronal FLAIR T2-weighted scan demonstrating confluent hyperintensity of the right frontotemporal cortex. FLAIR = fluid-attenuated inversion recovery.

When examining the supporting features in these 86 patients with a clinical diagnosis of MOGAD, children had more supporting features than adults, especially myelitis and optic neuritis (*p* < 0.0001) (Table 2). In particular, the highest median number of supporting features was seen in children presenting with transverse myelitis, followed by children with optic neuritis and adults and children with ADEM (Table 2). The most common supporting feature in children presenting with optic neuritis was longitudinal optic nerve involvement (81.3%), while in adults, it was bilateral simultaneous optic nerve involvement (50%) (Table 2, Figure 2). All children and adults with myelitis showed longitudinally extensive lesions, and these lesions were central (with the H sign) in all children, but only 12.5% of adults (Table 2, Figure 2). While a similar proportion of children (70%) and adults (80%) with brain, brainstem, or cerebral syndrome showed ill-defined T2 lesions, cortical lesions were seen in 22% of children, but in none in adults (Table 2, Figure 2). Patients with relapsing disease (N = 30) had more supporting criteria at follow-up than onset (median 3.5 vs 2, *p* = 0.03) and a higher number of supporting features than patients with monophasic disease at final follow-up (*p* = 0.016). The number of supporting features at onset was similar between patients with monophasic (N = 56) and relapsing (N = 30) disease. Children had a higher number of supporting features compared with adults (*p* = 0.0011, Mann-Whitney *U*).

**Table 2:** Clinical and Radiologic Supporting Features, Which Are Listed in the 2023 International Criteria for a Diagnosis of MOGAD, in 86 Patients With a Clinical Diagnosis of MOGAD

Supporting clinical or MRI features	Children presenting with optic neuritis (N = 16)	Adults presenting with optic neuritis (n = 24)	Children presenting with transverse myelitis (n = 6)	Adults presenting with transverse myelitis (n = 8)	Children presenting with brain, brainstem, or cerebral syndrome (n = 27)	Adults presenting with brain, brainstem, or cerebral syndrome (n = 5)
Female	11 (68.8%)	14 (58.3%)	2 (33.3%)	2 (25%)	15 (55.6%)	2 (40%)
No. of patients who relapsed over time	5 (31%)	9 (37.5%)	0	0	14 (51.8%)	3 (60%)
Optic nerve involvement	16 (100%)	24 (100%)	0	4 (50%)	4 (14.8%)	3 (60%)
Bilateral simultaneous clinical involvement	11 (68.8%)	12 (50%)	0	3 (37.5%)	1 (3.7%)	3 (60%)
Longitudinal optic nerve involvement	13 (81.3%)	10 (41.7%)	0	0	3 (11.1%)	3 (60%)
Perineural optic sheath enhancement	5 (31.3%)	6 (25%)	0	0	1 (14.8%)	0
Optic disc edema	12 (75%)	11 (45.8%)	0	1 (20%)	4 (14.8%)	2 (40%)
Spinal cord involvement	1 (6.3%)	4 (16.7%)	6 (100%)	8 (100%)	7 (25.9%)	2 (40%)
Longitudinally extensive myelitis	0	0	6 (100%)	8 (100%)	7 (25.9%)	2 (40%)
Central cord lesion or H-sign	0	2 (8.3%)	6 (100%)	1 (12.5%)	7 (25.9%)	2 (40%)
Conus lesion	0	1 (4.2%)	4 (66.7%)	2 (25%)	2 (7.4%)	1 (20%)
Brain, brainstem, or cerebral involvement	6 (37.5%)	0	5 (83.3%)	0	27 (100%)	5 (100%)
Ill-defined T2 hyperintense lesions in supratentorial and infratentorial white matter	5 (31.3%)	0	4 (66.7%)	0	19 (70.4%)	4 (80%)
Deep gray matter involvement	3 (18.8%)	0	4 (66.7%)	0	19 (70.4%)	3 (60%)
Lesions involving pons, middle cerebellar peduncle, or medulla	3 (18.8%)	0	3 (50%)	0	15 (55.6%)	1 (20%)
Cortical lesions ± leptomeningeal enhancement	2 (12.5%)	0	1 (16.7%)	2 (25%)	14 (21.9%)	0
Median number of supporting features per patient	3	2	4.5	1	3	3

Abbreviations: ADEM = acute disseminated encephalomyelitis; MOGAD = myelin oligodendrocyte glycoprotein antibody–associated disease.

Patients with a diagnosis of ADEM were included in the brain, brainstem, or cerebral syndrome category. The proportion of patients with specific supporting features of the total number of patients with each presenting phenotype was calculated.

Note: That adding the proportion does not get to 100% because some patients may have more than 1 supporting features within the same phenotype or across phenotypes.

The application of the 2023 international MOGAD criteria to the 539 patients who fulfilled the inclusion criteria diagnosed MOGAD in 88 patients; 83 patients (children 49, adults 34) had a prior clinical diagnosis of MOGAD and were considered true positive. The remaining 5 patients (1 child and 4 adults) were considered false positives, that is, they all had a final clinical diagnosis of relapsing-remitting MS and met the MOGAD criteria (i.e., they had a positive fixed-CBA result during their diagnostic workup and at least 1 supporting feature). These patients presented with optic neuritis (N = 3), transverse myelitis (N = 1), or a brainstem phenotype (internuclear ophthalmoplegia) (N = 1); they all showed oligoclonal bands in the CSF unmatched to the serum, brain MRI lesions that were considered suggestive of MS, and at least 1 supporting feature (longitudinal optic nerve involvement [N = 2], perineural sheath enhancement [N = 1], optic disc oedema on fundoscopy [N = 1], cortical lesion [N = 1], central cord lesion [N = 2], and conus lesion [N = 2]); with 1 patient having 3 supporting features, 2 patients having 2, and 2 patients having 1. There were 3 patients who did not meet MOGAD criteria but had a clinical diagnosis of MOGAD (false negative); they were all MOG-Ab–positive adults with optic neuritis (2 of whom had relapsing inflammatory optic neuropathy), normal brain and spinal cord MRI, and no supporting features. Four hundred forty-eight patients were considered as true negative. Among the 539 patients included into the analysis, there were 17 patients who were MOG-Ab positive but were not diagnosed with MOGAD according to clinical judgment of their neurologists; their clinical and paraclinical features are summarized in eTable 1. Demographics, length of follow-up, time to testing, and number of tests for patients positive vs negative for MOG-Abs, stratified in adults vs children, are summarized in eTable 2.

The 2023 international MOGAD diagnostic criteria showed excellent sensitivity (96.5%), more so in children than adults (100% vs 91.9%), high specificity (98.9%), similar in both groups (children 98.8%, adults 98.9%), and high accuracy (98.5%) (children 99.2%, adults 98.3%) (Table 3). Among all the performance indicators, the NPV reached the highest probability (99.3%) (children 100%, adults 99.2%) (Table 3). Compared with MOG-Ab testing alone, the 2023 MOGAD diagnostic criteria had significantly higher specificity (98.9% vs 96.3%, *p* = 0.0005) and a higher PPV (94.3% vs 83.5%) (Table 3); the improvement in specificity came with a slight, nonstatistically significant reduction in sensitivity (96.5% vs 100%, *p* = 0.08); the NPV was the same (99.3% vs 100%) (Table 3). When looking at the improved performance of the MOGAD criteria vs MOG-Ab testing alone, this was driven exclusively by the adult cohort (sensitivity 91.9% vs 100%, *p* = 0.08; specificity 98.9% vs 95.6%, *p* = 0.0005) because in children, there were no changes in any of the performance characteristics (Table 3).

**Table 3:** Performance of the MOG-Ab Seropositivity and the 2023 International MOGAD Criteria When Clinical MOGAD Diagnosis Was Used as Benchmarking Against Which the New Criteria Were Tested

	Sensitivity, % (95% CI)	Specificity, % (95% CI)	Accuracy, % (95% CIs)	PPV, % (95% CI)	NPV, % (95% CIs)
All patients					
MOG-Ab testing	100 (95.7–100)	96.3 (94.1–97.6)	96.9 (95.0–98.2)	83.5 (75.2–89.4)	100 (99.1–100)
2023 MOGAD diagnostic criteria	96.5 (90.2–99.1)	98.9 (97.4–99.5)	98.5 (97.1–99.4)	94.3 (87.4–97.6)	99.3 (98.1–99.8)
Children					
MOG-Ab testing	100 (92.7–100)	98.8 (93.7–99.9)	99.2 (95.9–100)	98.0 (89.5–99.9)	100 (95.7–100)
2023 MOGAD diagnostic criteria	100 (92.7–100)	98.8 (93.7–99.9)	99.2 (95.9–100)	98.0 (89.5–99.9)	100 (95.7–100)
Adults					
MOG-Ab testing	100 (90.69–100)	95.6 (93.0–97.3)	96.0 (93.7–97.2)	69.8 (56.5–80.5)	100 (98.9–100)
2023 MOGAD diagnostic criteria	91.9 (78.7–97.2)	98.9 (93.7–99.9)	98.3 (96.5–99.3)	89.4 (75.8–95.8)	99.2 (97.6–99.7)

Abbreviations: MOG-Ab = MOG antibody; MOGAD = MOG-Ab–associated disease; NPV = negative predictive value; PPV = positive predictive value.

## Discussion

We showed that the 2023 MOGAD diagnostic criteria performed very well in a large selected cohort of adults and children, followed up for a median of 3.6 years. The diagnosis based on best clinical judgment before the 2023 MOGAD criteria was used as benchmark against which the new criteria were tested. In our center, which relies on fixed CBA MOG-Ab assays, the 2023 MOGAD criteria differ from MOG seropositivity alone by the additional requirement of 1 or more clinical/radiologic supporting features. This may help to explain the improved specificity and positive predictive value, which were exclusively seen in our adult cohort.

Our results highlight important discussion points. The performance characteristics of the MOGAD criteria, and even of MOG-Ab testing alone, were generally very high. This may have multiple causes. First, specialist clinics in CNS demyelination are likely to have higher MOGAD prevalence than general neurology settings, especially for pediatric services (approximately 40% of all CNS demyelination diseases in children are MOGAD). The prevalence of MOG-Ab in the tested population affects both PPV and NPV because higher prevalence increases the PPV and can decrease the NPV. This implies that our findings can only be generalized to similar populations with a higher pretest probability of MOGAD. However, sensitivity and specificity are not influenced by the pretest probability of MOGAD, and these were still high with the new MOGAD criteria, especially in children.^6^

Second, as stated in the 2023 MOGAD criteria manuscript, MOG-Ab testing should not be used to screen all neurologic demyelinating presentations but should be selectively applied by experienced neuroimmunology specialists based on supporting features to enhance their PPV.^6^ This guidance for their use was also supported by the 2018 international recommendations for MOG-Ab testing in suspected CNS demyelination because MOG-Ab screening of relatively unselected cohorts may reduce the PPV of the test by increasing the false-positive rate.^9^ Similarly, the 2023 MOGAD consortium cautioned that universal screening with MOG-IgG in unselected populations with moderate/low pretest probability would lead to an intermediate/low PPV.^6^ Therefore, to conform to these 2 international consensus recommendations, we applied the 2023 MOGAD criteria to patients from specialized neuroimmunology clinics, demonstrating a higher pretest probability of MOGAD.^6,9^

Routine clinical practice is to test all children with acquired demyelinating syndrome for both MOG and AQP4 antibodies, and the high accuracy of the MOGAD criteria in this group, which is related to the high frequency of supporting features seen in pediatric cases, and the high pretest probability of MOGAD vindicate this approach. In our adult cohort presenting with a clinically isolated syndrome, particularly optic neuritis, we tested for MOG-Ab when there was diagnostic uncertainty or when we applied the McDonald MS diagnostic criteria,^10^ which requires the exclusion of alternative diagnoses; this approach aligns with general recommendations to test for MOG-Ab in patients with optic neuritis.^11^ Both the MS criteria and MOGAD criteria require exclusion of the other's diagnosis before their own application. This issue is consequent to the combined interpretation of both criteria sets that will require future resolution.^7^ The frequency of MOG-Ab in adults with unilateral optic neuritis ranges from 1.7% in the US optic neuritis treatment trial^12^ to 5% in a more recent US cohort^13^ and 20.2% in China.^14^ In binocular simultaneous optic neuritis, MOG-Ab seropositivity increases to 26.3%–66.7%.^14^ Only 5 patients (4 of whom were adults) fulfilled the MOGAD criteria (i.e., they were MOG-Ab positive and had at least 1 supporting feature) but had a diagnosis of MS (e.g., false positive). In cases with diagnostic uncertainty, other factors, such as repeat testing in both the serum and CSF,^15^ testing with live CBA, oligoclonal bands (which are more common in MS, although they can be seen in 20% of patients with MOGAD^16,17^), and lesion dynamics on MRI^18,19^ could be assessed over time to discriminate the demyelinating etiology, before commencing disease-modifying therapies.

Children had more supporting criteria than adults, especially in myelitis and optic neuritis phenotypes. Children, when showing a predominant brain, brainstem, or cerebral syndrome (including ADEM) fulfilled more supporting features than adults. This is unsurprising because an ADEM presentation is more common in the pediatric age group.^20^ The age-related clinical phenotype seen in MOGAD is well described with children younger than 10 years more likely to have brain lesions.^21,22^

Blinded, multicenter, international studies suggest that live CBAs are the gold standard for MOG-Ab testing^23,24^; however, these require cell culture facilities and can have a long turnaround time. As a result, an increased number of clinical laboratories are using the fixed CBAs routinely, as is the case in our centers. Fixed CBAs do not routinely provide titers and, therefore, require the supporting features to be present for the diagnosis of MOGAD. In our cohort, only adults with a diagnosis of MOGAD and CRION who had been on long-term immunosuppression did not fulfill these 2023 international criteria (e.g., false negative); this may be due to (1) bilateral optic nerve involvement, which is seen less frequently in adults than in children (2) a delay in time from symptom onset to clinical review in a tertiary center, which reduces the probability of seeing optic disc swelling, (3) lack of systematic imaging protocols to include dedicated orbit imaging with contrast to evaluate the length of the lesion and the enhancement patterns, and (4) the effect of immunosuppression. Standardized imaging protocols may be required for all patients presenting with optic neuritis to increase the sensitivity of the diagnostic criteria, especially in adults.^25^

A limitation of our study was that not all patients were seen by a neuroimmunology specialist during presentation, with some of the adult patients being seen and tested for MOG-Ab after their onset. This delay in MOG-Ab testing could have affected the performance of the criteria, as recently suggested.^26^ Patients were tested over a wide period post symptom onset and often after immunotherapy; it is therefore possible that patients who were tested after immunotherapy may have been seronegative then. It is of interest that relapsing patients had more supporting features at the last follow-up. This may suggest that in patients who do not fulfill the diagnostic criteria for MOGAD at their first attack, the diagnosis may become clearer with time and after multiple relapses. MOG-Ab titers were not available to us. If they had been available and greater than 1:100, we could have observed an increased sensitivity of the MOGAD criteria because the 3 current false-negative cases (i.e., patients who did not meet MOGAD criteria, but had a clinical diagnosis of MOGAD) could have become true positive. We did not test patients for coexisting MOG IgM and IgA antibodies. An earlier report suggested that these antibodies occur in 23/120 (19%) patients with MOGAD and in 2/114 (1.7%) patients with seronegative demyelinating diseases and are not clinically relevant.^27^ A more recent study identified MOG-IgA in 3/50 (6%) patients with seronegative CNS demyelination and suggested they may be a useful diagnostic marker,^28^ but this requires further study. We also did not test MOG-Ab in the CSF, which is associated with a small, additional pickup rate over the serum alone.^15^ Patients with MOGAD had more antibody testing than patients without MOGAD. This is because there remains an unanswered clinical question regarding the role of persistent MOG-Ab serostatus and the risk of relapse. In contrast to a recent study reporting that 4/61 patients tested MOG-Ab negative at symptom onset,^29^ none of the patients with MOGAD reported here tested negative initially. In patients in whom MOG-Ab were negative, repeat testing was only performed if there was high clinical suspicion or if the patient relapsed. It is not a common practice to regularly test MOG-Ab negative patients. Furthermore, for many of the patients with suspected MOGAD at onset, an alternative diagnosis became clearer later, thereby precluding the need for repeat testing.

Finally, one should note potential repercussions of requiring MOG-Ab seropositivity to diagnose MOGAD, as recommended by the criteria. This predication has arisen historically, resulting from the recognition of certain clinical syndromes being associated with MOG seropositivity, and is in contrast to the evolution of AQP4-Ab diagnostic criteria, which has more secure grounding in laboratory science.^30^ There are several implications. First, only patients with clinical and paraclinical characteristics suggestive of MOGAD should be tested, which implies a necessary selection bias if the criteria are due to be applied as recommended. Future work should address the inherent selection bias required by the diagnostic criteria. Second, with a pivotal role of MOG seropositivity in the diagnosis of MOGAD, seronegative MOGAD does not currently exist as an entity. Consequently, any confusion matrix depending on MOG seropositivity will be heavily skewed toward very high sensitivities and NPVs because MOGAD with absent MOG-Abs cannot be diagnosed. In addition, the role of MOG seropositivity as a neuroinflammatory marker also needs refinement. For example, it is unclear how transient or fluctuating MOG seropositivity, sometimes seen with repeated serum sampling even off treatment, should be applied to MOGAD diagnostic criteria. In addition, MOG-Abs have been reported in conditions considered noncore by the 2023 MOGAD criteria, such as orbital inflammatory disease^31,32^ and thymic hyperplasia,^33^ so how does one decide whether to include them in future or is the MOG-Ab association spurious for noncore presentations? Furthermore, there are unresolved issues about the role of MOG seropositivity in patients diagnosed with MS. Further basic science and translational research is needed to fully understand the role of MOG-Ab in MOGAD. Nevertheless, the 2023 MOGAD diagnostic criteria have formalized a diagnostic paradigm that can standardize clinically related MOGAD research to address these outstanding issues.

Although the international panel did not recommend routine testing of MOG-Ab and did not support the application of the criteria to patients with a better alternative diagnosis (i.e., MS), we have applied the 2023 MOGAD diagnostic criteria to a selected cohort of patients tested for MOG-Abs and found that these 2023 international MOGAD criteria still exhibit high performance. This was reflected by an improvement above relying on MOG-Ab seropositivity alone in the adult cohort, largely driven by the presence of supporting features, suggesting that it is a crucial criterion for MOGAD in adults.

## Appendix Authors

**Table:**

Name	Location	Contribution
James A. Varley, DPhil	Queen Square MS Centre, Department of Neuroinflammation, UCL Queen Square Institute of Neurology, University College London; Department of Brain Sciences, Charing Cross Hospital, Imperial College London, United Kingdom	Drafting/revision of the article for content, including medical writing for content; major role in the acquisition of data; study concept or design; and analysis or interpretation of data
Dimitrios Champsas, MBBS	Department of Neurology, Great Ormond Street Hospital for Children, London, United Kingdom	Drafting/revision of the article for content, including medical writing for content; major role in the acquisition of data
Timothy Prossor, MD	The National Hospital for Neurology and Neurosurgery, UCLH NHS Trust, London, United Kingdom	Drafting/revision of the article for content, including medical writing for content; major role in the acquisition of data
Giuseppe Pontillo, MD, PhD	Queen Square MS Centre, Department of Neuroinflammation, UCL Queen Square Institute of Neurology, University College London, London, United Kingdom	Analysis or interpretation of data
Omar Abdel-Mannan, MD	Queen Square MS Centre, Department of Neuroinflammation, UCL Queen Square Institute of Neurology, University College London; Department of Neurology, Great Ormond Street Hospital for Children, London, United Kingdom	Drafting/revision of the article for content, including medical writing for content; major role in the acquisition of data
Zhaleh Khaleeli, MD	Queen Square MS Centre, Department of Neuroinflammation, UCL Queen Square Institute of Neurology, University College London; The National Hospital for Neurology and Neurosurgery, UCLH NHS Trust, London, United Kingdom	Drafting/revision of the article for content, including medical writing for content; major role in the acquisition of data
Axel Petzold, MD, PhD	Queen Square MS Centre, Department of Neuroinflammation, UCL Queen Square Institute of Neurology, University College London; The National Hospital for Neurology and Neurosurgery, UCLH NHS Trust, London, United Kingdom; Neuro-ophthalmology Expert Centre, Amsterdam UMC, Amsterdam, the Netherlands; Moorfields Eye Hospital NHS Foundation Trust, London, United Kingdom	Drafting/revision of the article for content, including medical writing for content; major role in the acquisition of data
Ahmed T. Toosy, FRCP	Queen Square MS Centre, Department of Neuroinflammation, UCL Queen Square Institute of Neurology, University College London; The National Hospital for Neurology and Neurosurgery, UCLH NHS Trust, London, United Kingdom	Drafting/revision of the article for content, including medical writing for content; major role in the acquisition of data; and analysis or interpretation of data
Sachid A. Trip, BSc, MBChB, PhD, FRCP	Queen Square MS Centre, Department of Neuroinflammation, UCL Queen Square Institute of Neurology, University College London; The National Hospital for Neurology and Neurosurgery, UCLH NHS Trust, London, United Kingdom	Drafting/revision of the article for content, including medical writing for content; major role in the acquisition of data; and study concept or design
Heather Wilson, MD	Queen Square MS Centre, Department of Neuroinflammation, UCL Queen Square Institute of Neurology, University College London; The National Hospital for Neurology and Neurosurgery, UCLH NHS Trust, London, United Kingdom	Drafting/revision of the article for content, including medical writing for content; major role in the acquisition of data
Dermot H. Mallon, BSc, MB ChB, MSc, PhD, FRCR	Queen Square MS Centre, Department of Neuroinflammation, UCL Queen Square Institute of Neurology, University College London; The National Hospital for Neurology and Neurosurgery, UCLH NHS Trust, London, United Kingdom	Drafting/revision of the article for content, including medical writing for content; major role in the acquisition of data
Cheryl Hemingway, MD, PhD	Queen Square MS Centre, Department of Neuroinflammation, UCL Queen Square Institute of Neurology, University College London; Department of Neurology, Great Ormond Street Hospital for Children, London, United Kingdom	Drafting/revision of the article for content, including medical writing for content; major role in the acquisition of data
Kshitij Mankad, MBBS	Department of Radiology, Great Ormond Street Hospital for Children, London, United Kingdom	Major role in the acquisition of data
Michael Kin Loon Chou	Neuroimmunology and CSF Laboratory, National Hospital for Neurology and Neurosurgery, London, United Kingdom	Major role in the acquisition of data
Andrew J. Church, PhD	Neuroimmunology and CSF Laboratory, National Hospital for Neurology and Neurosurgery, London, United Kingdom	Major role in the acquisition of data
Melanie S. Hart, PhD	Queen Square MS Centre, Department of Neuroinflammation, UCL Queen Square Institute of Neurology, University College London; The National Hospital for Neurology and Neurosurgery, UCLH NHS Trust; Neuroimmunology and CSF Laboratory, National Hospital for Neurology and Neurosurgery; National Institute for Health and Care Research (NIHR) University College London Hospitals Biomedical Research Centre, United Kingdom	Drafting/revision of the article for content, including medical writing for content; major role in the acquisition of data; and analysis or interpretation of data
Michael P. Lunn, MA, MBBS, FRCP, PhD	The National Hospital for Neurology and Neurosurgery, UCLH NHS Trust; Neuroimmunology and CSF Laboratory, National Hospital for Neurology and Neurosurgery; National Institute for Health and Care Research (NIHR) University College London Hospitals Biomedical Research Centre; Department of Neuromuscular Diseases, UCL Queen Square Institute of Neurology, University College London, United Kingdom	Drafting/revision of the article for content, including medical writing for content; major role in the acquisition of data; and analysis or interpretation of data
Wallace Brownlee, MBChB	Queen Square MS Centre, Department of Neuroinflammation, UCL Queen Square Institute of Neurology, University College London; The National Hospital for Neurology and Neurosurgery, UCLH NHS Trust; National Institute for Health and Care Research (NIHR) University College London Hospitals Biomedical Research Centre, United Kingdom	Drafting/revision of the article for content, including medical writing for content; major role in the acquisition of data; study concept or design; and analysis or interpretation of data
Yael Hacohen, MD, PhD	Queen Square MS Centre, Department of Neuroinflammation, UCL Queen Square Institute of Neurology, University College London; Department of Neurology, Great Ormond Street Hospital for Children, London, United Kingdom	Drafting/revision of the article for content, including medical writing for content; major role in the acquisition of data; study concept or design; and analysis or interpretation of data
Olga Ciccarelli, MD, PhD, FRCP	Queen Square MS Centre, Department of Neuroinflammation, UCL Queen Square Institute of Neurology, University College London; The National Hospital for Neurology and Neurosurgery, UCLH NHS Trust; National Institute for Health and Care Research (NIHR) University College London Hospitals Biomedical Research Centre, United Kingdom	Drafting/revision of the article for content, including medical writing for content; major role in the acquisition of data; study concept or design; and analysis or interpretation of data

## Glossary

ADEM: acute disseminated encephalomyelitis
AQP4-Ab: aquaporin-4 antibody
CBA: cell-based assay
MOG: myelin oligodendrocyte glycoprotein
MOG-Ab: MOG antibody
MOGAD: MOG-Ab–associated disease
MS: multiple sclerosis
NMOSD: neuromyelitis optica spectrum disorder
NPV: negative predictive value
PPV: positive predictive value
